# Semisynthetic Glycoconjugate Vaccine Candidates against *Cryptococcus neoformans*

**DOI:** 10.1021/acsinfecdis.4c00094

**Published:** 2024-05-31

**Authors:** Conor J. Crawford, Livia Liporagi-Lopes, Carolina Coelho, Samuel R. Santos Junior, André Moraes Nicola, Maggie P. Wear, Raghav Vij, Stefan Oscarson, Arturo Casadevall

**Affiliations:** † Centre for Synthesis and Chemical Biology, University College Dublin, Belfield, Dublin D04 V1W8, Ireland; ‡ Department of Molecular Microbiology and Immunology, Johns Hopkins Bloomberg School of Public Health, 615 North Wolfe Street, Baltimore 21205, Maryland, United States

**Keywords:** Cryptococcus neoformans, World Health Organization, glucuronoxylomannan (GXM), clinical cryptococcosis, glycoconjugates produced

## Abstract

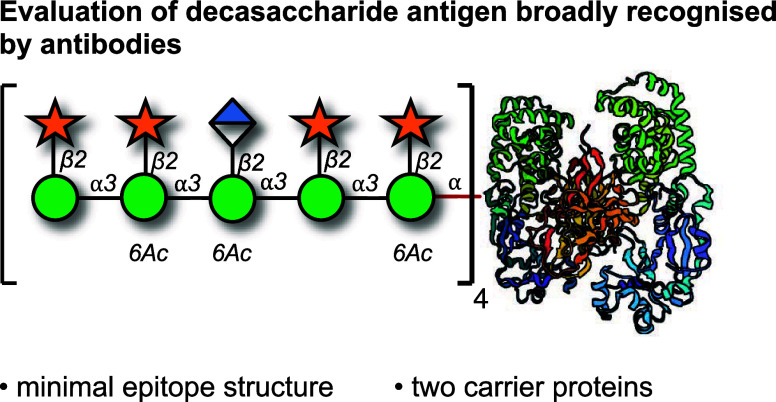

*Cryptococcus neoformans* is a fungus classified by the World Health Organization as a critically important pathogen, which poses a significant threat to immunocompromised individuals. In this study, we present the chemical synthesis and evaluation of two semisynthetic vaccine candidates targeting the capsular polysaccharide glucuronoxylomannan (GXM) of *C. neoformans*. These semisynthetic glycoconjugate vaccines contain an identical synthetic decasaccharide (M2 motif) antigen. This antigen is present in serotype A strains, which constitute 95% of the clinical cryptococcosis cases. This synthetic oligosaccharide was conjugated to two proteins (CRM197 and Anthrax 63 kDa PA) and tested for immunogenicity in mice. The conjugates elicited a specific antibody response that bound to the M2 motif but also exhibited additional cross-reactivity toward M1 and M4 GXM motifs. Both glycoconjugates produced antibodies that bound to GXM in ELISA assays and to live fungal cells. Mice immunized with the CRM197 glycoconjugate produced weakly opsonic antibodies and displayed trends toward increased median survival relative to mice given a mock PBS injection (18 vs 15 days, *p* = 0.06). These findings indicate promise, achieving a successful vaccine demands further optimization of the glycoconjugate. This antigen could serve as a component in a multivalent GXM motif vaccine.

*Cryptococcus neoformans* is an environmentally ubiquitous fungus that can cause cryptococcosis in immunocompromised patients, such as solid-organ transplant recipients or individuals with AIDS.^1^ Infection often occurs undetected and the fungus can remain dormant in the host for decades.^1^ If immunosuppression occurs, the latent infections can re-emerge and potentially cause lethal meningitis. Current therapy necessitates the use of antifungal drugs for months, and even with such treatments, high mortality is observed. Furthermore, clinicians are observing rising drug resistance to antifungal drugs.^2,3^ Therefore, better therapeutic and preventative strategies against cryptococcosis are required. The urgency of this issue was recognized by the World Health Organization (WHO), which created a fungal priority pathogens list, with *C. neoformans* in the top critical priority group.^4^

Vaccination holds promise in preventing disease and eliminating the necessity for prolonged and costly antifungal treatments. A prophylactic vaccine could be used to safeguard at-risk groups such as solid-organ transplant patients. A common strategy used in *C. neoformans* vaccine development has been to utilize antigens present on the fungal cell surface, including glycolipids, glycoproteins, and polysaccharides.^5−11^ The primary component of the *C. neoformans* capsular polysaccharide is glucuronoxylomannan (GXM), which is essential for virulence.^12,13^ This polysaccharide does not contain a defined repeating unit but rather contains discrete motifs that co-occur which results in enormous antigenic complexity (SI Figure 1).^14,15^ It is currently not possible to make a chemically defined vaccine by isolating homogeneous oligosaccharide fragments from the capsule or shed polysaccharide. Consequently, chemical synthesis is the only option available for testing the antigenicity of polysaccharide motifs. To induce T-cell activation mechanisms aiming for long-term immunity across diverse populations, including those with compromised immune systems, a polysaccharide must be conjugated to a carrier protein.^16^

Such glycoconjugate vaccine approaches have been tested using GXM.^10,17^ These conjugates were made utilizing heterogeneous fungal polysaccharides^10,14,17^ and cyanogen bromide activation, an imprecise conjugation method that generates cross-linked matrices. In vivo, these conjugate vaccines were found to exhibit batch-to-batch variability, which may be attributed to the structural diversity of both the polysaccharides and the resulting conjugates.^8,10^

Carbohydrate chemistry allows the creation of well-defined material.^18^ These synthetic glycans allow detailed structure–function studies, which are required to obtain a deeper biomolecular understanding that enables rational vaccine design. For example, we recently reported the first solution structure of a cryptococcal oligosaccharide.^19^ Potential benefits include enhanced immunogenicity, improved specificity, and standardized composition. Synthetic carbohydrate antigens have been successfully used to develop commercial vaccines against *Haemophilus influenzae* type b and are under clinical investigation against *Shigella*.^20,21^ Synthetic carbohydrate antigens, including β-glucans,^22,23^ and β-mannans,^24,25^ have been tested in mice as candidate vaccines against fungi.^22−25^ A synthetic heptasaccharide human serum albumin (HSA) conjugate vaccine against *C. neoformans* was found to induce an IgG immune response in mice.^11^ These antibodies recognized fungal cells in a punctuate pattern,^11^ but monoclonal antibodies (mAbs) derived from the spleens of immunized mice were not opsonic and did not protect mice in challenge experiments.^9^

Subsequent research, utilizing GXM microarrays,^26,27^ molecular modeling,^28^ and NMR spectroscopy,^19^ has revealed that larger M2 motif GXM oligosaccharides are required to adopt the conformations of GXM polysaccharides. Furthermore, these conformations are important for epitope presentation, enabling binding by both protective and nonprotective mAbs.^26^ This means that the previously studied heptasaccharide M2 motif antigen was perhaps too small to assume solution-phase confirmations of GXM polysaccharides, offering a hypothesis for its poor efficacy in vivo,^9^ and that this heptasaccharide structure is rarely recognized by mAbs on microarray surfaces.^26^ In contrast, decasaccharide **1** (Figure 1) has been shown to mimic the M2 motif in GXM polysaccharides,^19^ and is widely recognized by protective and nonprotective IgG and IgM mAbs on microarrays.^26^

**Figure 1:**
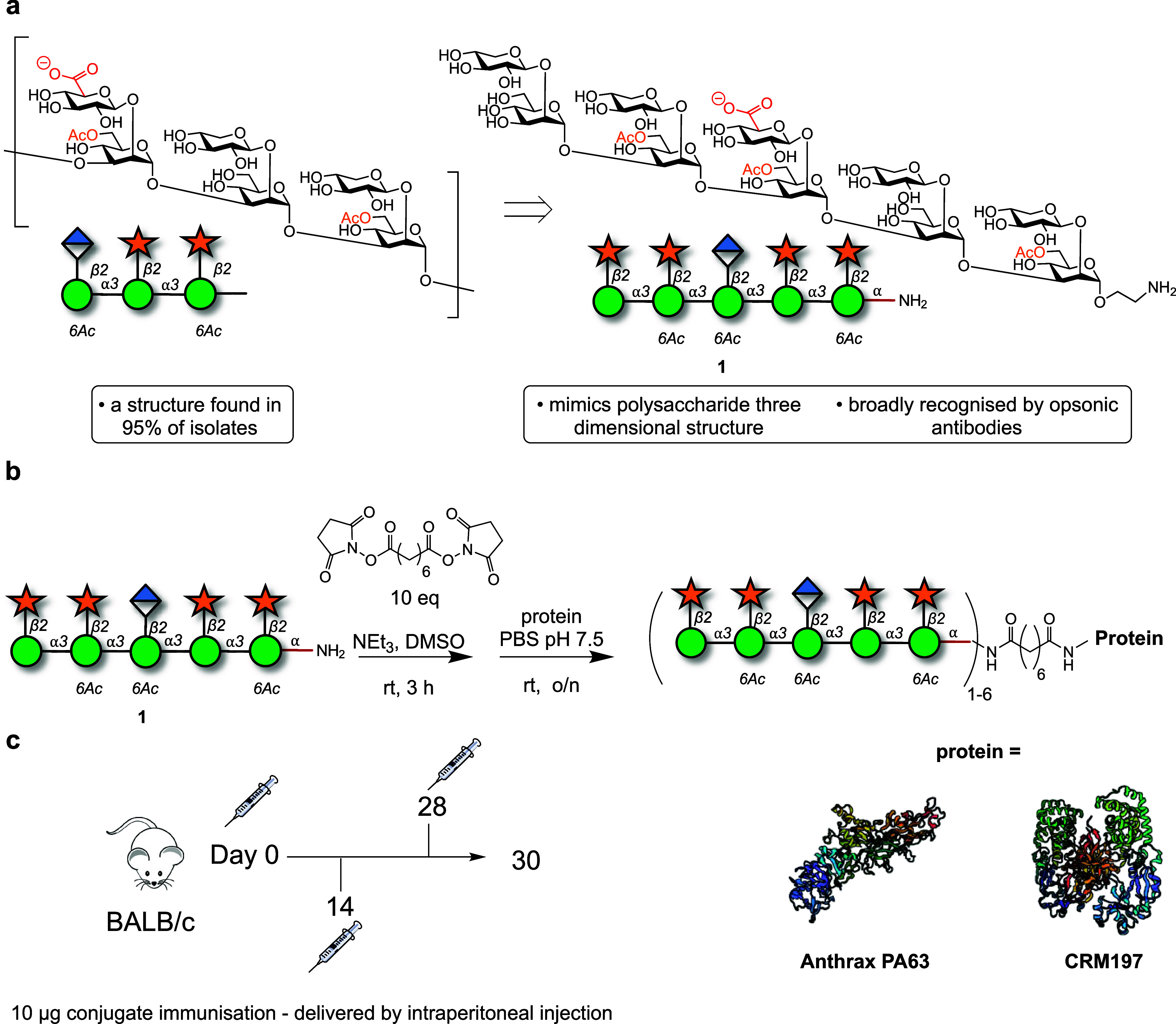
Synthesis and evaluation of two semisynthetic vaccine candidates targeting the capsular polysaccharide, glucuronoxylomannan (GXM), of *C. neoformans*. (a) Structure of motif 2 of GXM and the decasaccharide antigen. (b) Conjugation of glycan **1** to carrier proteins anthrax PA63 or CRM197. (c) Immunization schedule.

This suggested that decasaccharide **1** could be a more effective oligosaccharide antigen in vaccination.^19,27,28^ Here, we report two vaccine candidates featuring the decasaccharide M2 motif but conjugated to two distinct carrier proteins: cross-reactive material 197 (CRM197) and anthrax protective antigen fragment 63 kDa (PA63). These conjugates were evaluated for their immunogenic properties in mice.

## Results and Discussion

### Synthesis of Semisynthetic Glycoconjugate Vaccine Candidates

Synthetic decasaccharide **1** was synthesized via a convergent approach employing thioglycoside building blocks.^27,29−31^ Following synthesis, glycan **1** underwent a reaction with an excess of a bis-NHS-activated suberic acid spacer. The intermediate product was then purified and conjugated to either CRM197 or PA63. MALDI-TOF analysis verified the complete conversion of each carrier protein into glycoconjugates, with a loading range of 1–6 glycans per protein (Figure 1, Table 1 and SI Figure 2).

**Table 1:** Loading of DECA-Protein Conjugates

entry	antigen	protein	MW range of conjugate (kDa)	average glycan loading per protein loading
1	**1**	CRM197	60–69	4
2	**1**	PA63	66–76	4

### Immunization with Synthetic Decasaccharide-protein Conjugates Elicits Antibodies That Bind GXM

Mice (*n* = 5 control groups/PBS and *n* = 10 immunized groups) were intraperitoneally immunized with 10 μg of decasaccharide-PA66 (DECA-PA63) or the decasaccharide-CRM197 (DECA-CRM197) conjugates in complete Freund’s adjuvant, followed by two interval boosts 14 days apart (with 10 μg of conjugate in incomplete Freund’s adjuvant on days 14 and 28) (Figure 1c).

**Figure 2:**
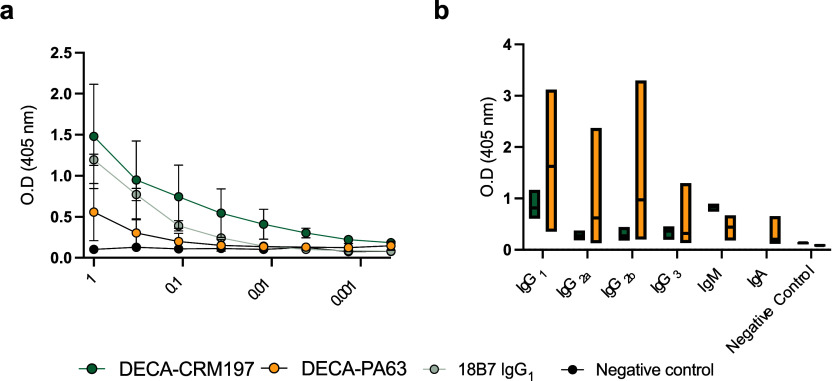
(a) GXM ELISA of immunized mouse serum to EPS. For panel (a) each dot represents the mean, and bars represent standard deviation. A positive control of mAb 18B7 known to react with both GXM standards, and a negative control of PBS were used for the ELISA. (b) Isotype distribution of antibodies found in sera from the DECA-CRM197 and DECA-PA63 conjugates. For isotype determination, Indirect ELISA with CPS coating, followed by sera, and then antibodies of differing isotypes as indicated. All mice show predominantly IgG1 and IgM isotypes. For panel (b) the middle line in each bar indicates the mean, range is min to max. The serum for GXM reactivity and isotype analysis were isolated by retroorbital bleeding after the full immunization protocol. Experiments were repeated in triplicate.

The immune serum reactivity to GXM exopolysaccharide (EPS) confirmed a GXM-binding antibody response in mice after the third immunization in the DECA-CRM197 and DECA-PA63 groups, while no such response was observed in the control groups (Figure 2). The DECA-CRM197 sera bound to *C. neoformans* EPS with a similar affinity and decay pattern as mAb 18B7 (IgG1) (Figure 2a). This serum was further examined for binding to capsular polysaccharide (CPS)-coated ELISA plates, and in contrast to the EPS-ELISA plates, mAb 18B7 showed a higher binding affinity (SI Figure 3). This suggests potential differences in the epitope composition of CPS and EPS preparations (SI Figure 3). Competition ELISA with serum from DECA-CRM197 mice vs mAbs 18B7 (IgG1), 13F1 (IgM), and 2D10 (IgM) found that the DECA-CRM197 serum reduced binding of all three mAbs, suggesting competition for the same epitopes (SI Figure 4).

### Isotype Composition of Antibodies in Sera from Glycoconjugate Vaccine Immunized Mice was Carrier Protein-dependent

Isotyping analysis of the immune sera from both conjugate vaccine immunizations uncovered a carrier protein-dependent difference. The immunoglobulin response was more consistent in DECA-CRM197 immunized mice compared to those immunized with the DECA-PA63 conjugate, with the latter showing a greater range of affinity and isotypes (Figure 2b). The CRM197 immunized mice produced a predominance of IgG_1_ and IgM isotype classes with lower levels of IgG2a, IgG2b and IgG3 detected. In the DECA-PA63 immunized mice, high levels of IgG1 were also detected, and high levels of the IgG2b subclass. The DECA-PA63 mice produced lower levels of IgM, IgG2a and IgA. These results highlight the role of carrier protein selection.^32^

### Immunization with GXM M2 Motif Elicits Cross-reactive Sera

**Figure 3:**
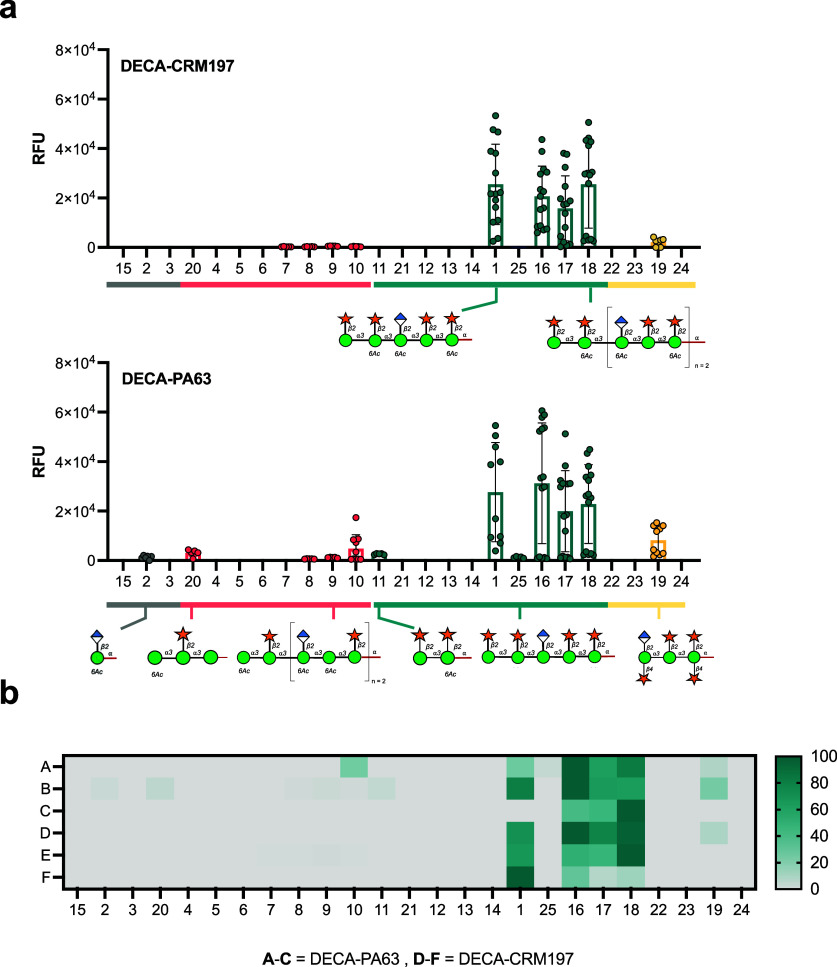
Microarray reactivity analysis of sera from mice immunized with CRM197 and PA63 glycoconjugates toward oligosaccharides. (a) Pooled response of DECA-CRM197 and DECA-PA63 mice. (b) A heatmap of comparing glycan binding profiles of sera from both conjugates. Rows A–C are DECA-PA63 mice, and rows D–F are DECA-CRM197 mice. The heatmap is normalized by lowest and highest point in each data set. The full library of the oligosaccharides structure can be found in Figure 4. A data point is the RFU of a single glycan spot on the array.

The molecular reactivity of the antibodies elicited during immunization was analyzed using a GXM microarray by selecting three mice randomly from each group.^26^ The conjugates predominantly elicited an immune response specific to the M2 motif of *O*-acetylated glycans (**1**, **16**–**18**, and **25**), with comparatively weak reactivity to other GXM motifs. A single mouse out of the six immunized with the DECA-PA63 conjugate elicited antibodies manifested reactivity toward the non-*O*-acetylated decasaccharide **25** (Figure 3b, row A). No binding to M2 motif glycans smaller than the decasaccharide **1** was observed in the DECA-CRM197 mice. In contrast, the DECA-PA63 conjugate elicited antibodies with reactivity toward two smaller structures: the first, a disaccharide of glucuronic acid-β-(1,2)-linked to 6-*O*-acetylated mannose (**2**), a motif found in the center of the decasaccaride antigen; the second, tetrasaccharide **11**, the terminal epitope of the decasaccaride antigen (Figure 3a).

Cross-reactivity toward the M4 and M1 GXM motifs was elicited by both GXM conjugates. The CRM197 and PA63 conjugates both resulted in reactivity toward glycan **19**, a nonacetylated octasaccharide representative of the M4 motif. Additionally, the glycoconjugates elicited additional reactivity toward the M1 motif, for the DECA-PA63 mice recognizing structures **8**, **9**, **10**, and **20**. The DECA-CRM197 sera has reactivity toward M1GXM motif glycans **7**–**10** (Figure 3). A single mouse from the DECA-PA63 group (total *n* of mice = 3) did not recognize decasaccharide **1** on the microarray but did show reactivity toward larger M2 motif glycans **16**, **17**, and **18** (Figure 3b, row C). These results demonstrate heterogeneity in the specificity of the immune response, even among genetically identical mice.

The binding reactivities of mAbs 18B7, 13F1, and 2D10 were previously determined by GXM microarray, indicating a predominant affinity toward the M2 motif of GXM.^26^ Moreover, 18B7 and 13F1 displayed additional reactivity toward the M4 motif, and 18B7 demonstrated the ability to bind to the M1 motif. Therefore, the microarray analysis supports the findings of the competition ELISA (SI Figure 4), suggesting that the elicited antibodies could compete for M1, M2, and M4 motifs.

**Figure 4:**
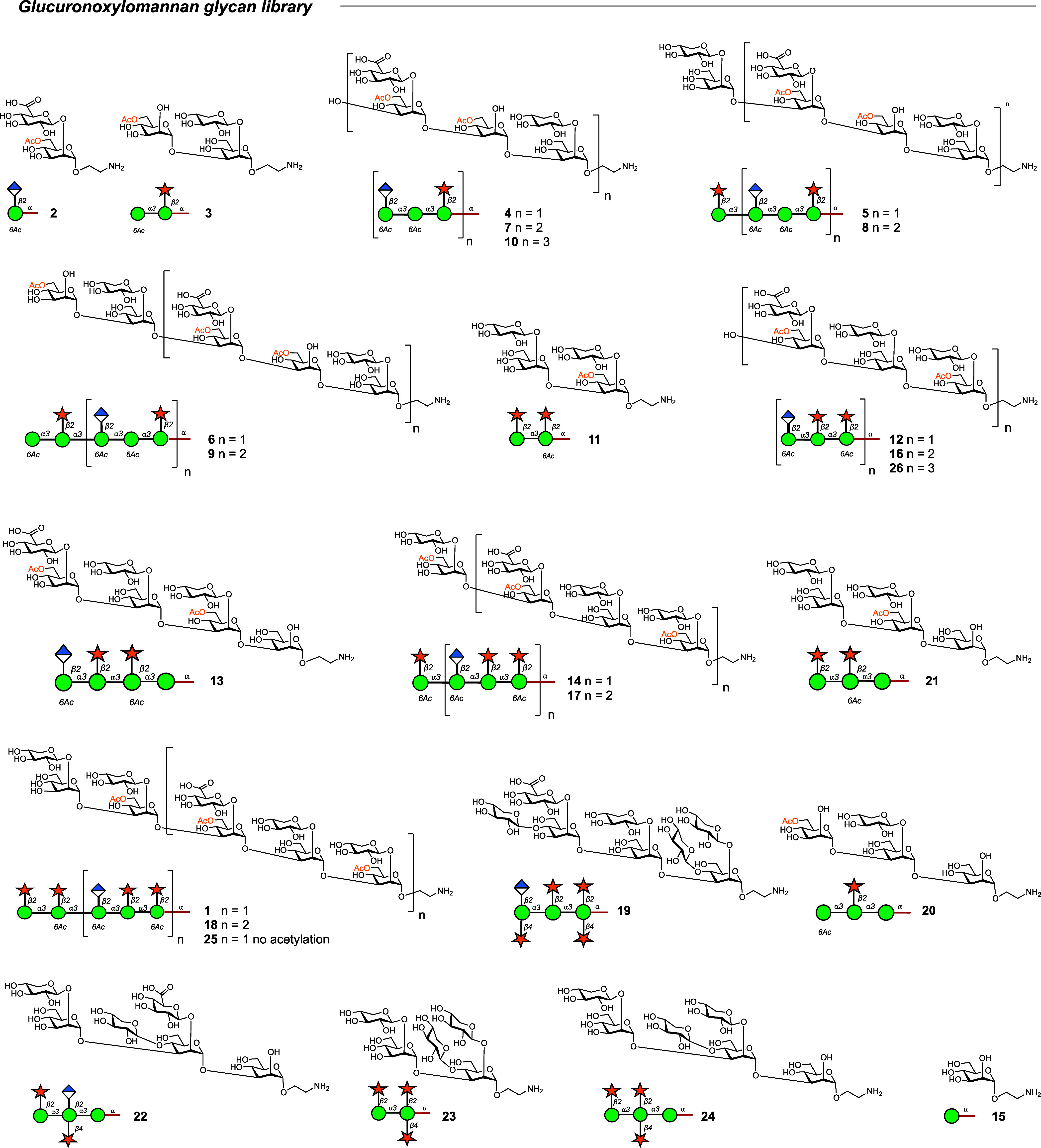
Glucuronoxylomannan glycan microarray library. Full library of the oligosaccharide GXM structures.

The low cross-reactivity observed in mice immunized with a single GXM motif conjugate suggests that all GXM motifs, when present in large oligosaccharides, share common structural epitopes. This is evident from the rare binding to GXM M2 oligosaccharides smaller than decasaccharide (**1**), and the fact that cross-reactivity between motifs only initiates at the decasaccharide (**7**) level for M1 motifs and at the octasaccharide stage for GXM M4 motifs (Figures 3 and 4). In future vaccine candidates, it would be interesting to elicit high reactivity toward all GXM motifs. Incorporating several oligosaccharides into a multivalent conjugate may achieve this.

### Semisynthetic Glycoconjugates Elicit Antibodies That Bind to *C. neoformans* Capsules

The capsule of *C. neoformans* is highly antigenically diverse and is characterized by a series of GXM motifs (SI Figure 1).^33^ These motifs exhibit dynamic variations both spatially, in their proximity to the cell wall, and temporally, evolving over the fungus’s lifecycle.^34^ The binding patterns of antibodies to the capsule have implications for the protective and nonprotective efficacy of monoclonal antibodies.^35^

**Figure 5:**
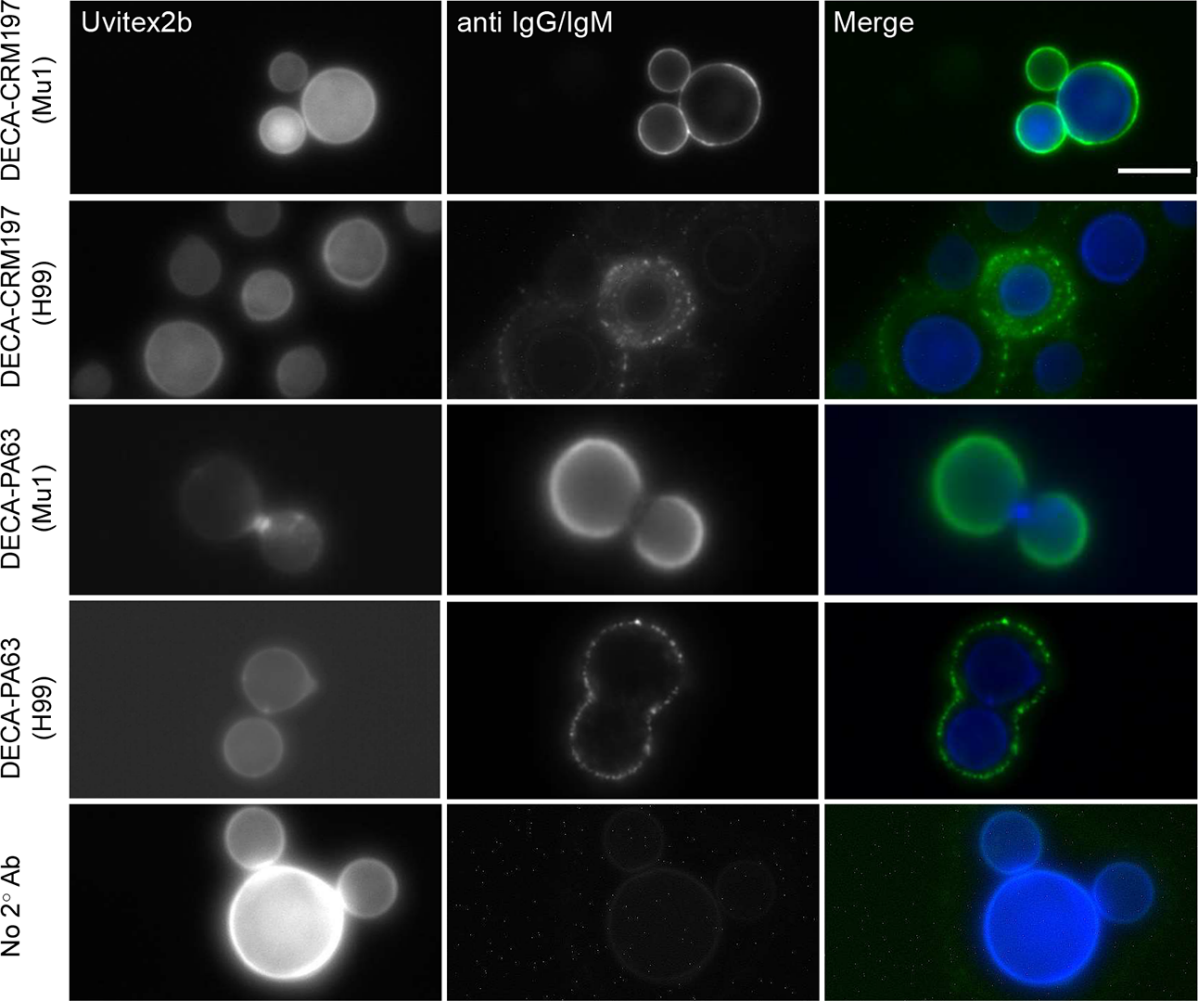
Antibodies from semisynthetic conjugates bind to *C. neoformans* cells. Mu1 and H99 were cultured for 48 h in capsule-inducing media and immunofluorescence was performed using the mouse serum from mice treated with 10 μg DECA-CRM197 or 10 μg DECA-PA63 conjugate. Cells were imaged for Uvitext2b (Blue channel) and secondary antibodies (IgG and IgM) (Green channel). Left margin indicates conjugate and inside the parentheses is the strain of *C. neoformans*. Merged images show annular and punctate staining in immunized mouse serum dependent on strain. Scale bar: 10 μM.

We tested the sera obtained after the immunization protocol for live imaging of fungal cells. For immunofluorescence, we utilized two strains of *C. neoformans*: H99, a serotype A lab strain known to express multiple GXM motifs, and Mu-1, also serotype A, which exclusively expresses the M2 motif of GXM.^14^ The serum binding to H99 fungal cells from the DECA-PA63 conjugate showed low consistency (8%, 4 positive cells, 49 cells total), with the majority of cells showing no staining. In contrast, the DECA-CRM197 conjugate elicited antibodies that stained the majority of cells (80%, 20 positive cells, and 24 cells total) (Figure 5). Sera from both conjugates exhibited a punctate pattern when binding to H99 while binding to the Mu-1 strain displayed an annular pattern.^36^

As the conjugates primarily are reactive to the M2 motif (Figure 3), it suggests that achieving annular binding to more antigenically diverse capsules requires antibodies with both diverse and high affinity binding. This may be obtained by immunization with several GXM motifs.

### DECA-CRM197 Conjugate Vaccine Elicits Weakly Opsonic Antibodies

**Figure 6:**
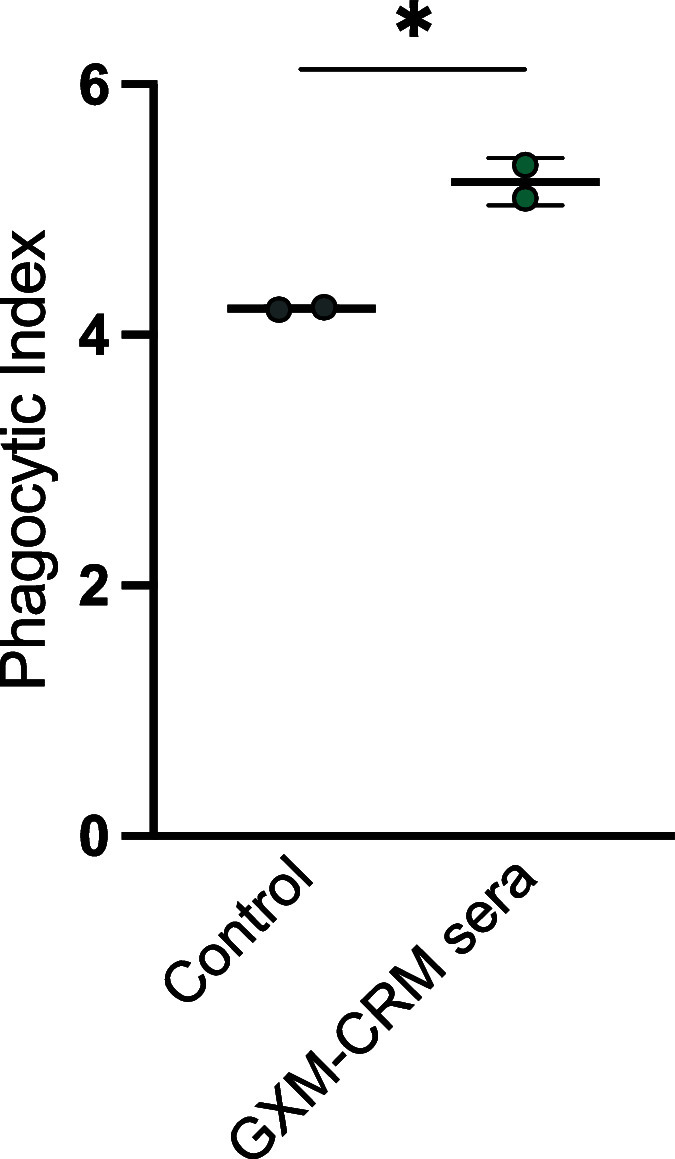
DECA-CRM197 conjugate vaccine elicited opsonic antibodies. The dot represents an individual experiment; the line is the mean, and error bars are standard deviation (SD). BMDM-derived macrophages were cocultured with *C. neoformans* strain 24067 in the presence of 20% guinea pig serum (as a control) and 1:50 dilution of serum from 10 μg immunized mice. Cells were counted after 2 h by using microscopy with a 40× objective. The phagocytic index (MOI 3:1) was determined by the number of internalized cryptococcal cells per 100 macrophages.

The capacity of antibodies to promote phagocytosis by immune cells is linked to protection in cryptococcosis,^37^ but not all opsonic antibodies are protective in mouse models of infection. The DECA-CRM197 sera was subject to further investigation for its ability to phagocytose fungal cells because of (i) its higher binding by ELISA (Figure 2a) and (ii) the microarray analysis suggested that these sera bound to conformational epitopes, which has been shown to be crucial for mAbs known to be opsonic (Figure 3a). (iii) and its more reliable binding to fungal cells by immunofluorescence (Figure 5). The sera derived from DECA-CRM197 immunization increased phagocytosis by BMDM cells (1:50 dilution) (*, *p* = 0,0169, unpaired *t*-test) against *C. neoformans* compared to the control group (20% (v/v) guinea pig serum) (Figure 6). However, this effect was modest but does differ in the DECA-CRM197 conjugate from the previous heptasaccaride-HSA conjugate.^9,11^ This improvement may arise due to (i) differences in the glycan antigens or (ii) the use of a more immunogenic carrier protein.

The increase in opsonic efficacy for vaccine immune sera relative to the control was relatively small, but it is important to note that opsonic antibodies are not necessarily protective. This is evidenced by the efficiency of a nonprotective IgG3 to GXM as an opsonin.^38^ Finally, it is important to consider that while phagocytosis is often inferred as a sign of efficacy, the fungus has been shown to be able to replicate inside macrophages and exit without causing cell lysis. However, it can also lead to cell destruction, either lytically or nonlytically.^39,40^

### Immunization DECA-CRM197 had Modest or No Effect On Survival Time

The immunization protocol was repeated (10 μg × 3, days 14, 21, 28) and on day 42 the mice (*n* = 5 control groups and *n* = 10 immunized groups) were challenged with 1.0 × 10^7^ yeasts of *C. neoformans* strain H99 or KN99-α.

**Figure 7:**
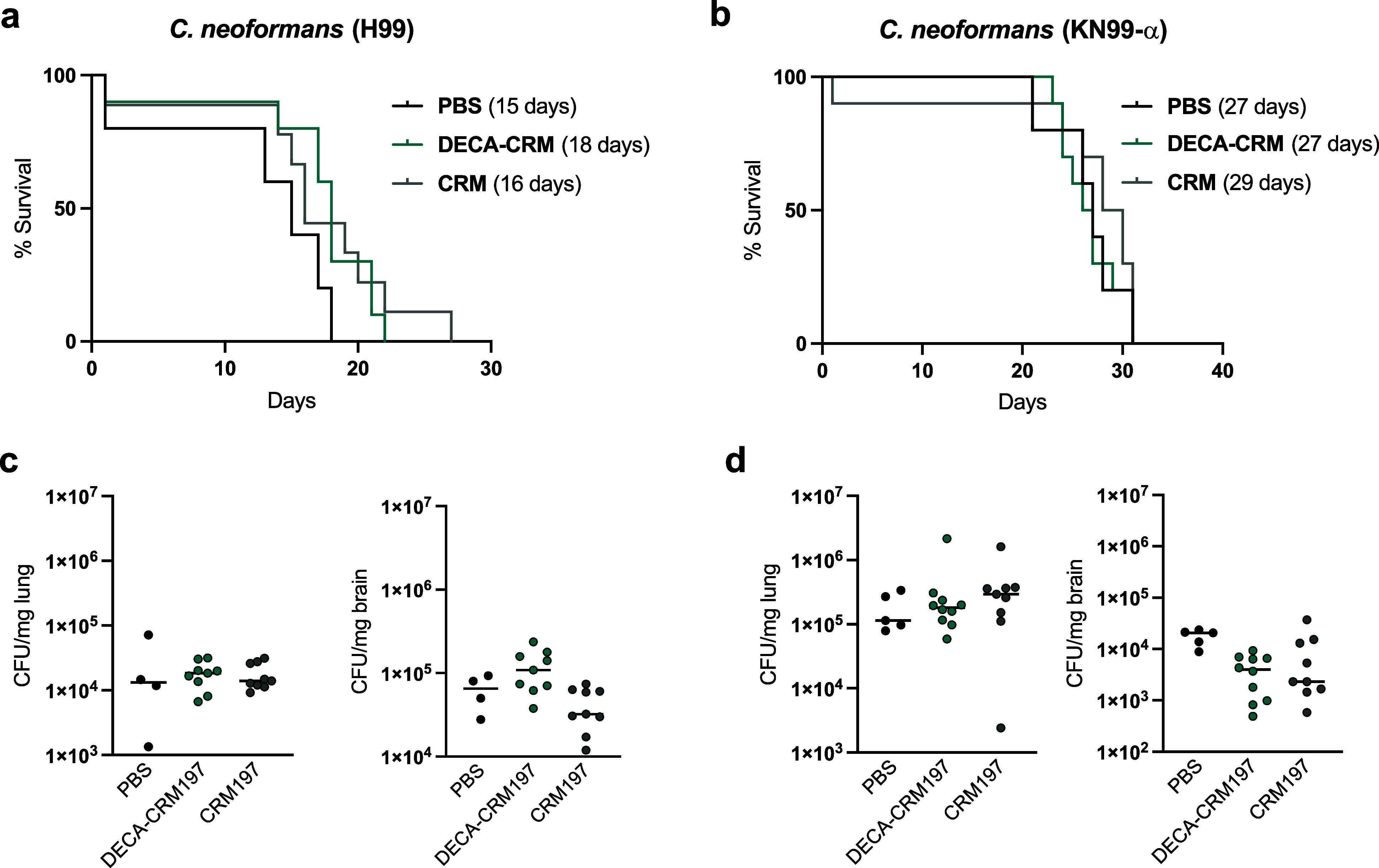
Challenge experiments with DECA-CRM197 conjugates against two strains of *C. neoformans*. (a) H99 challenge and (b) KN99-α challenge, in parentheses, is the median survival time. (c) Lung and brain fungal burden at day of the mouse death via CFU for H99. (d) Lung and brain fungal burden at day of the mouse death via CFU for KN99-α challenge. Shown is the median with individual points showing CFU per tissue/mouse.

Mice challenged with *C. neoformans* H99 had the longest median survival time when they received the DECA-CRM197 conjugate (18 days). Followed by those immunized with CRM197 (16 days), those given a PBS mock infection had the lowest median survival time (15 days; Figure 7a). Comparison of mice immunized with DECA-CRM197 to those given a mock PBS infection was just above significance (*p* = 0.0678, Kaplan–Meier survival analysis Log-rank (Mantel-Cox) test).

Post-challenge, all mice were analyzed for colony-forming units (CFUs) in the lung and the brain, which are organs known to be involved in systemic cryptococcosis. Finding that CFUs in the brain of H99-challenged mice show a significant difference between groups (**, *p* = 0.0052, Kruskal–Wallis test). CRM197 immunized mice were found to have lower CFUs compared to the DECA-CRM197 mice (**, *p* = 0.0078, posthoc Dunn’s multiple comparisons test) (Figure 7c), raising the possibility that some of the effects observed in mice reflected a protective effect to the carrier protein. Analysis of the colony-forming units (CFUs) in the lungs showed no difference between groups (nanoseconds, *p* = 0.8217, Kruskal–Wallis test).

Mice challenged with strain KN99-α showed no difference in survival (*n* = 5 control groups and *n* = 10 immunized groups, Kaplan–Meier survival analysis), with CRM-immunized mice having the highest median survival time at 29 days and mice in both the DECA-CRM197 and PBS having median survival times of 27 days (Figure 7b).

In the KN99-α challenged mice, the groups showed distinct response in the brain (*, *p* = 0.0193, Kruskal–Wallis test) with the DECA-CRM197 immunized mice having lower CFUs compared to control groups (*, *p* = 0.0172, post-Dunn’s test), while in the lungs, KN99-α-challenged mice showed no distinct response between groups (ns, *p* = 0,3898, Kruskal–Wallis test) (Figure 7d).

## Conclusions

We report the synthesis and evaluation of two semisynthetic vaccine candidates targeting *C. neoformans*, both incorporating a decasaccharide (DECA) antigen but utilizing different carrier proteins (CRM197 and PA63). Mice exhibited a carrier protein-dependent immune response, with the DECA-CRM197 conjugate demonstrating a more defined and therefore more consistent outcome in mice. The DECA-CRM197 conjugate induced primarily IgG1 and IgM antibodies, while the DECA-PA63 conjugate induced IgG1 and IgG2b antibodies. Glycan microarray analysis revealed that both conjugates predominantly elicited sera reactivity toward the M2 motif, with concurrent weak cross-reactivity with the M1 and M4 motif. Sera from immunized mice had the ability to bind to *C. neoformans* H99 cells in a punctate pattern, which is associated with nonprotective antibodies in murine challenge studies, but did bind in annular patterns to the M2 motif capsule of Mu-1 cells.^35^ The DECA-CRM197 immunized mice sera were weakly opsonic.

Furthermore, despite promising aspects, the challenge experiment revealed only a modest increase in the median survival time against strain H99, with no statistical significance against KN99-α. Potential factors contributing to this outcome include inadequate levels of protective antibodies, low protective efficacy of the antibody response, and uncertainties around optimal glycan-protein loading, formulation, and immunization schedules. The antibodies of immunized mice bound to H99 fungal cells in a punctate manner and sometimes not at all, implying that most cells would likely escape the immune response. This may account for the modest protection observed. Overall, the DECA-CRM197 conjugate elicited antibodies that demonstrated modest in vitro functional activity compared to a previous heptasaccharide vaccine,^11^ but the efficacy results still fall short of those reported with glycoconjugates using cryptococcal GXM.^41^

The previous heptasaccaride-BSA conjugate, with an average loading of seven, and the decasaccaride-CRM197 and PA63 conjugates both with average loadings of 4, did not elicit a protective response in mice. Since some of the epitopes recognized by protective mAbs in the capsular polysaccharide seem to be conformational,^42^ we hypothesized that by synthesizing a larger oligosaccharide we would increase the likelihood of presenting such conformations to the immune system. However, the results of this study show that increasing the carbohydrate motif from seven to ten residues was not sufficient to elicit a protective response.

It is possible that a more comprehensive immunological evaluation of a range of glycoconjugates with different loadings may reveal an optimum loading where immunogenicity is maximized without masking or interfering with the recognition of important epitopes on the carrier protein. However, at this time we believe that given the complexity of structures recognized by protective mAbs,^26,27^ a more fruitful avenue for synthetic oligosaccharide vaccine research would be to develop a multivalent oligosaccharide-protein conjugate that would deliver multiple antigenic capsular motifs to the immune system. The DECA-CRM197 conjugate could serve as a valuable component in future multivalent vaccines aimed at preventing cryptococcosis.

## Methods

### Oligosaccharide Synthesis

Chemical synthesis of decasaccharide **1** followed published procedures.^27,29,30,43,44^ We used a convergent synthesis of di- and tetrasaccharide thioglycoside building blocks, which were coupled using dimethyl(methylthio)sulfonium trifluoromethanesulfonate (DMTST) in diethyl ether.^45^ A 2-naphthylmethyl (NAP) ether-protecting group was employed as a temporary protecting group to allow the extension of the oligosaccharide from the nonreducing end. Cleavage of the NAP ether was completed using 2,3-dichloro-5,6-dicyano-1,4-benzoquinone (DDQ) with a buffered aqueous component to suppress cleavage of benzyl ethers.^29^ Deprotection of the GXM used a preconditioned palladium on carbon catalyst which gave a selective catalyst for hydrogenolysis of the aromatic protecting groups to yield the desired decasaccharide **15**.^29,46^

### Conjugations

Decasaccharide **1** (1 equiv) was coupled to a bis(*N*-hydroxysuccinimide ester) suberic acid linker (5 equiv) in DMSO, which was followed by MALDI-TOF (SuperDHB, acetonitrile:water – 1% TFA, 50:50 v/v). Once complete, 5**1** was precipitated by the addition of ethyl acetate and centrifuged at 4 °C for 5 min. The supernatant was removed and washed twice with cold ethyl acetate to remove excess suberic acid linker. The precipitate was then resuspended in PBS (100 mM, pH 7.5) and added in 30-fold excess to either a solution of CRM197 or PA 63 PBS (100 mM, pH 7.5) and left overnight at room temperature (30 glycan: 1 protein). Conjugates were followed and analyzed by MALDI-TOF spectrometry in linear mode to determine the extent of conjugation (*trans*-ferulic acid, ethanol: water – 0.5% TFA, 50:50 v/v). Once satisfactory loading was achieved, the reaction mixture was transferred to a Vivaspin 500 centrifugal filter (MWCO 10 kDa, GE Healthcare, Buckinghamshire, UK), desalted, and washed three times with sterile water (MIKRO 200R, Hettich, 15 min at 13,000 rpm, 3 × 500 μL water). The conjugate was suspended in 1 mL of sterile water, and the final concentration of protein was determined using the extinction coefficient (ε) ε0.1% 1.07 for a 1 mg/mL at 280 nm (UV-1280 Shimadzu spectrometer) to yield 2.0 mg of the conjugate. Finally, the conjugate was lyophilized to give a white solid.

### Ethics Statement

All animal procedures were performed with prior approval from the Johns Hopkins University (JHU) Animal Care and Use Committee (IACUC), under approved protocol number MO18H152. Mice were handled and euthanized with CO_2_ in an appropriate chamber followed by thoracotomy as a secondary means of death in accordance with guidelines on Euthanasia of the American Veterinary Medical Association. JHU is accredited by AAALAC International, in compliance with Animal Welfare Act regulations and Public Health Service (PHS) Policy, and has a PHS Approved Animal Welfare Assurance with the NIH Office of Laboratory Animal Welfare. JHU Animal Welfare Assurance Number D16-00173 (A3272-01). JHU utilizes the United States Government laws and policies for the utilization and care of vertebrate animals used in testing, research, and training guidelines for appropriate animal use in a research and teaching setting.

### Microorganisms and Growth Conditions

*C. neoformans* serotype A strains H99 (ATCC 208821) and Kn99α were used in the DECA-CRM197 challenge experiments. The yeast cells were kept frozen in 10% glycerol. Sabouraud dextrose broth (SAB, from Gibco) medium was used for the standard growth of yeast cells at 30 °C with moderate shaking (120 rpm) overnight. For the immunofluorescence experiments, after the preculture on SAB medium, *C. neoformans* cells were transferred and grown for 3 days at 30 °C/120 rpm in minimal media (15 mM dextrose, 10 mM MgSO_4_, 29.4 mM KH_2_PO_4_, 13 mM glycine, and 3 μM thiamine-HCl), to maximize capsule development.

*C. neoformans* serotype D strain 24067 was used in the DECA-CRM197 sera phagocytosis experiments. The yeast cells were kept frozen in 10% glycerol. Sabouraud dextrose broth (SAB, from Gibco) medium was used for standard growth of yeast cells at 30 °C with moderate shaking (120 rpm) overnight, after the preculture on SAB medium cells were transferred and grown for 3 days at 30 °C/120 rpm in minimal media (15 mM dextrose, 10 mM MgSO_4_, 29.4 mM KH_2_PO_4_, 13 mM glycine, and 3 μM thiamine-HCl), to maximize capsule development.

### Cell Culture

NSObcl2 cells were obtained from the Albert Einstein College of Medicine hybridoma facility and maintained in RPMI supplemented with 10% fetal calf serum and 1 μg/mL G418.^47^ Hybridomas generated as described below were cultivated in the same medium.

### Mice and Immunization

Six-week-old female A/J mice were used to perform the immunizations. Mice were immunized intraperitoneally with 10 μg of DECA-PA63 or DECA-CRM197 in complete Freund’s adjuvant (Sigma, St. Louis, MO). Mice were boosted at 14-day intervals or as required with 10 μg of each conjugate in incomplete Freund’s adjuvant.^9^ Mice were bled by retro-orbital bleeding using heparin capillary tubes under isoflurane anesthesia, 2 weeks after immunizations.

### Serological Assays

Immune sera were assayed for their reactivity against GXM by an indirect Enzyme-Linked Immunosorbent Assay (ELISA). Exopolysaccharides (EPS) obtained by ultrafiltration from *C. neoformans* strain H99 cultures in minimal media grown for 3 days in a concentration of 10 μg/mL, were dissolved in PBS used to coat polystyrene plates (Corning *9018) by incubating overnight at 4 °C. CPS isolation and ELISA plates were created following published protocols.^48^ After blocking with 1% BSA in PBS, dilutions of the sera from the immunized mice were incubated for 1 h at 37 °C and then overnight at 4 °C. As a secondary antibody, we used alkaline phosphatase-conjugated goat antimouse IgG, IgA, and IgM (Southern Biotech) in a 1:1000 dilution, as indicated in figure legends, for 1 h at 37 °C. In other experiments, we used as secondary antibodies isotype-specific (IgA, IgG1, IgG2a, IgG2b, IgG3) alkaline-phosphatase-conjugated antibodies (Southern Biotech). Bound antibodies were detected using p-nitrophenyl phosphate (Sigma) as a substrate, with the absorbance measured in a plate spectrophotometer at 405 nm. For competition ELISA assays, mAbs 18B7, 13F1, and 2D10 were biotinylated with a biotin commercial kit, according to the manufacturer’s instructions (Pierce, Rockford, IL, USA). ELISA plates were generated as described above. After blocking, a constant concentration of the biotinylated mAb was incubated with decreasing concentrations of a different nonbiotinylated mAb in the blocking buffer for 1 h at 37 °C. After washing, avidin conjugated with alkaline phosphatase (Sigma-Aldrich) was added, and the preparation was incubated for 1 h at 37 °C. Absorbance at 405 nm was recorded after the reaction was developed with *p*NPP.^49^

### Glycan Microarray Scanning

The glycan microarray scanning was carried out as described.^50^ Primary anti-GXM mAbs or control Abs were prepared from stocks to the necessary concentration in 3% BSA in PBS-T. Biotinylated goat anti-mouse kappa chain Abs were used as secondary reagents for all primary antibodies. Detection was performed with the streptavidin-conjugated SureLight P3 fluorophore (Cayman Chemical Company, Ann Arbor, MI) at 5 μg/mL in PBS-T. All hybridization steps were performed using the Agilent 8-well gasket system in a humidity-controlled rotating hybridization oven at 26 °C for 1–2 h. Washes (X3) in Tris-buffered saline (pH 7.6, 0.1% Tween 20) (TBS-T) for 3 min and once for 3 min in TBS. Scanning was performed in an Agilent SureScan Dx microarray scanner with red wavelength emission detection. The data was processed on Mapix software. The mean fluorescent intensities (corrected for mean background) and standard deviations (SD) were calculated (*n* = 6).

### Phagocytosis Assay

Phagocytosis assay was performed as described: BMDM were seeded (5 × 10^4^ cells/well) on poly-d-lysine coated coverslip bottom MatTek Petri dishes with a 14 mm microwell (MatTek Brand Corporation). Cells were then incubated at 37 °C with 10% CO_2_ overnight. On the following day, BMDMs were infected with Uvitex 2B (Polysciences, Warrington, PA) stained *Cryptococcus neoformans* strain 24067 (1.5 × 10^5^ cells/well), and with the addition of sera (1:50 (v/v)) from three different mice of the same immunization group and/or complement (20% (v/v)) guinea pig serum – Fisher Scientific #642831). After 2 h of incubation to allow phagocytosis, the culture was washed five times with fresh medium to remove extracellular cryptococcal cells. In addition, Alexa fluor 568 (Thermo Fisher Scientific) conjugated to 18B7 mAb was added to stain the remaining extracellular fungal cells. Images were taken using an Olympus AX70 microscope (Olympus, Center Valley, PA) with a 40× objective. The *C. neoformans*/macrophage ratio was 3:1. The phagocytic index was determined by the number of internalized cryptococcal cells per 100 macrophages.^51^

### Immunofluorescence

Approximately 2.5 × 10^5^ cells/mL in 100 μL of blocking solution (PBS-1% BSA) was incubated with sera at a dilution of 1:50 for 30 min at RT. Cells were washed twice in a blocking solution. 1:100 of secondary antibodies; Goat anti-rat IgM (1 mg/mL), Goat anti-mouse kappa IgG FITC (1 mg/mL), Rabbit anti-goat IgG FITC (1 mg/mL), and Goat anti-mouse IgM (1 mg/mL), and 0.1 μg/mL Uvitex 2B, were added to the cells. Cells were imaged using an Olympus AX70 microscope.

### Survival Study

Mice (*n* = 7 animals in control groups and *n* = 8 animals treated groups) were immunized three times (day 0, day 14, and day 28) as follows: (1) a group was injected intraperitoneally only with CRM197 (control group #1); (2) a group was injected intraperitoneally with DECA-CRM197 conjugate; and (3) a group was injected intraperitoneally with PBS (control group #2). All immunogens were emulsified in Freund’s adjuvant (first one with complete adjuvant; second and third ones with incomplete adjuvant). Mice were challenged intranasally with 1.0 × 10^7^*C. neoformans* cells per animal (KN99- or H99)^52^ 14 days after the last immunization. The animals were observed daily for 30 days and euthanized if they showed more than 20% weight loss or inability to feed.

### Fungal Burden Assessment

The fungal burden was evaluated at the time of death by counting CFU (colony-forming units). The lungs and brain were removed, weighed, and homogenized in 1 mL of PBS. After serial dilutions, homogenates were inoculated on Sabouraud agar plates with 1% streptomycin/penicillin (Corning, NY). The plates were incubated at room temperature, and the colonies were counted after 48–72 h.

### Statistics

Statistical analyses were done using GraphPad Prism version 8.00 for Mac OS X (GraphPad Software, San Diego, CA, USA). Statistical analyses for the survival analysis used Kaplan–Meier with Log-rank (Mantel–Cox test) and Gehan–Breslow–Wilcoxon tests. One-way analysis of variance using a Kruskal–Wallis nonparametric test was used to compare the differences between groups, and individual comparisons of groups were performed using Dunn’s multiple comparisons test.

## Supporting Information Available

GXM motifs (M1, M2, and M4), demonstrating the antigenic diversity found in the capsule of *C. neoformans*; MALDI-TOF of glycoconjugates DECA-CRM197 and DECA-PA63; ELISA with capsular polysaccharide and DECA-CRM197 conjugates; and competition ELISA between mAbs and sera (PDF)
