# Supplementary material for: Semisynthetic Glycoconjugate Vaccine Candidates against Cryptococcus neoformans
Source: ACS Infect Dis. 2024 May 31;10(6):2089–100. doi: 10.1021/acsinfecdis.4c00094 (PMC11184550; doi:10.1021/acsinfecdis.4c00094)
Supplement: Supplementary file 1 — id4c00094_si_001.pdf [file id4c00094_si_001.pdf]

Supporting Information

Semi-synthetic glycoconjugate vaccine candidates against *Cryptococcus neoformans*

Conor J. Crawford,[1,2,3] Livia Liporagi-Lopes,[2,4] Carolina Coelho,[2,6] Samuel R. Santos Junior,[2] André Moraes Nicola,[2,5] Maggie P. Wear,[2] Raghav Vij,[2,7] Stefan Oscarson,[1*] Arturo Casadevall[2*]

[1]Centre for Synthesis and Chemical Biology, University College Dublin, Belfield, Dublin, D04 V1W8, Ireland, [2]Department of Molecular Microbiology and Immunology, Johns Hopkins Bloomberg School of Public Health 615 North Wolfe Street, Baltimore, MD 21205, USA, [3]Present Address: Max Planck Institute for Colloids and Interfaces, Am Mühlenberg1, 14476 Potsdam, Germany.[4]Present Address: Departamento de Análises Clínicas e Toxicológicas, Faculdade de Farmácia, Universidade Federal do Rio de Janeiro, Rio de Janeiro, 21941-902, Brazil, [5]Present Address: Faculty of Medicine, University of Brasília, Brasília, 70910-900, Brazil, [6]Present Address: MRC Centre for Medical Mycology, University of Exeter, Exeter Devon, EX4 4QD, UK. [7] Present address: Leibniz Institute for Natural Product Research and Infection Biology, Jena, 07745, Germany

Email: * stefan.oscarson@ucd.ie and * acasade1@jhu.edu

Supporting Figures

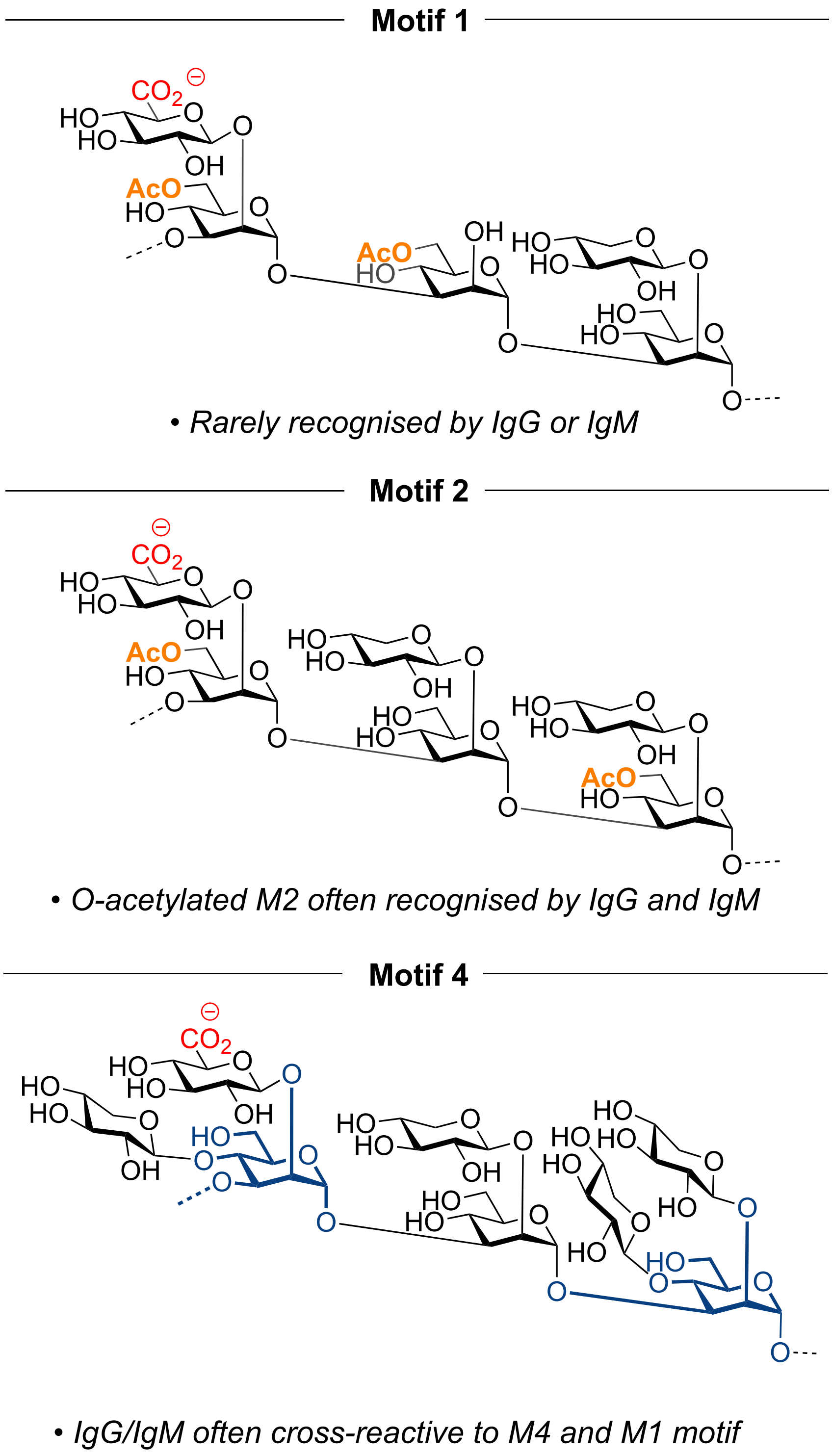

SI Figure 1. GXM motifs. GXM motifs (M1, M2 and M4), demonstrating the antigenic diversity found in the capsule of *C. neoformans*.

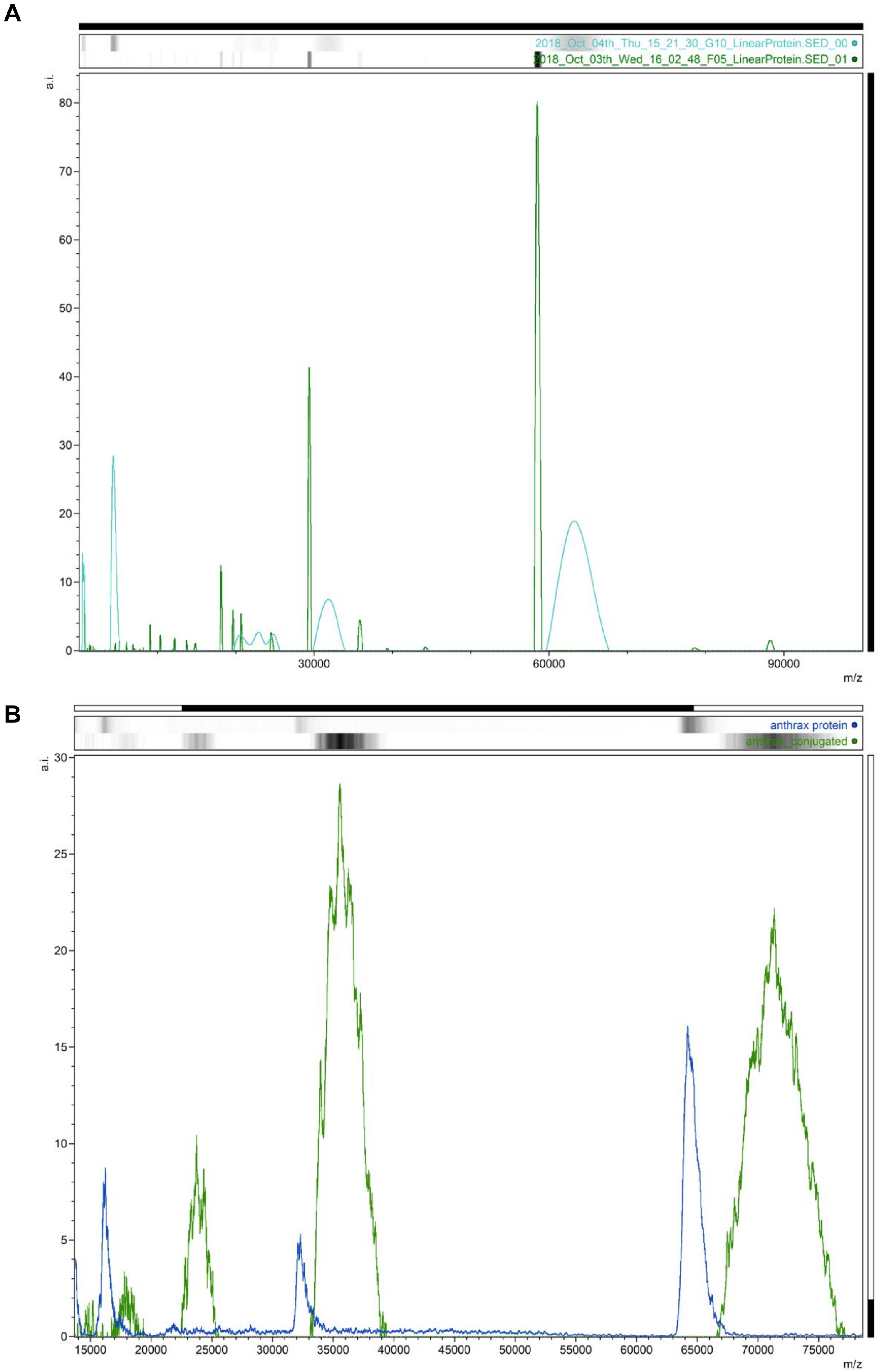

SI Figure 2. MALDI-TOF of glycoconjugates DECA-CRM197 and DECA-PA63. A. DECA-CRM197, shown in green is CRM197 and in blue DECA-CRM197. **b** DECA-PA63 conjugate, shown in green is DECA-PA63 and blue is PA63.

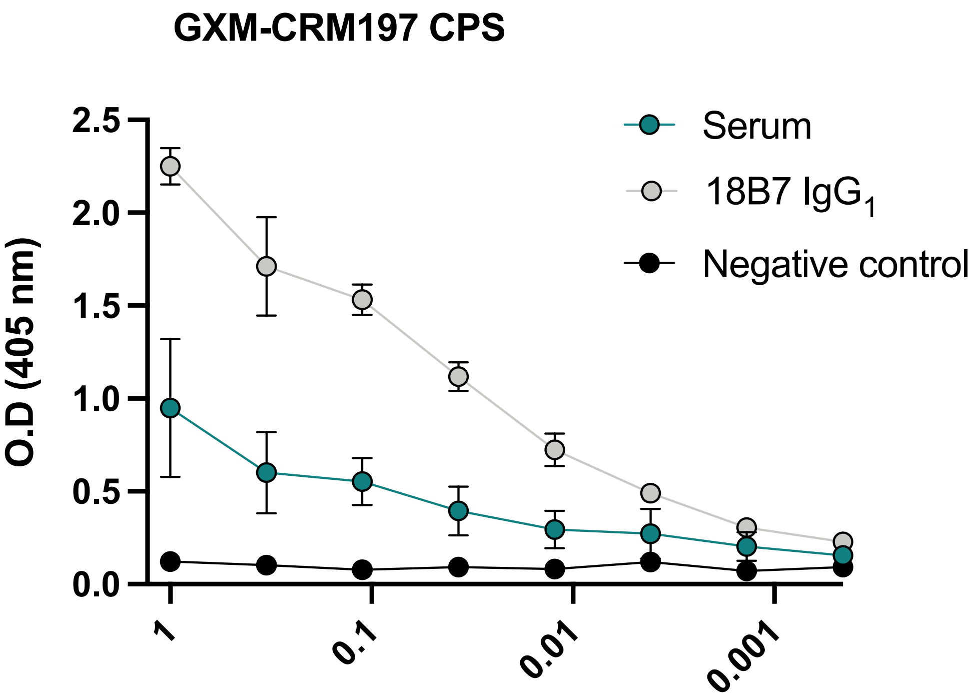

SI Figure 3. ELISA with capsular polysaccharide and DECA-CRM197 conjugates. Serum from DECA-CRM197 was examined for binding to CPS-coated ELISA plates and exhibited a higher affinity. Positive control: mAb 18B7; Negative control: irrelevant mAb.

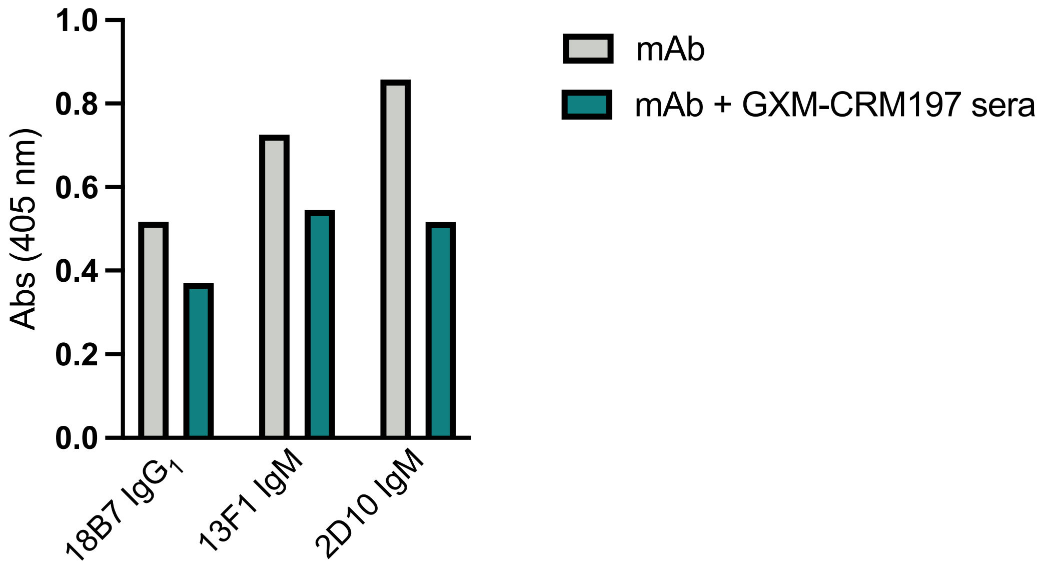

SI Figure 4. Competition ELISA between mAbs and sera. Serum from DECA-CRM197 mice vs mAbs 18B7 (IgG1), 13F1 (IgM) and 2D10 (IgM) showed that all three mAbs could compete for the same epitopes.
